# A study of factors influencing Chinese college students’ intention of using metaverse technology for basketball learning: Extending the technology acceptance model

**DOI:** 10.3389/fpsyg.2022.1049972

**Published:** 2022-12-20

**Authors:** Longfei Ren, Fangfang Yang, Chao Gu, Jie Sun, Yunfeng Liu

**Affiliations:** ^1^Department of Sports Science, Honam University, Gwangju, South Korea; ^2^Department of Culture and Arts Management, Honam University, Gwangju, South Korea; ^3^College of Arts and Design, Zhejiang Agriculture and Forest University, Hangzhou, China; ^4^Department of Physical Education of Linfen College, Shanxi Normal University, Linfen, China

**Keywords:** metaverse, basketball teaching, TAM, virtual, attitude

## Abstract

**Introduction:**

Based on the expansion of flow constructs based on the TAM model, this study assesses the impact of metaverse technology in college basketball courses.

**Methods:**

We surveyed 849 effective samples using an online questionnaire survey, verified our analysis using structural equation modeling, and examined the moderating effect of gender on the path relationship.

**Results:**

The perceived ease of use, the flow experience, and the perceived usefulness of the product are important predictors of behavioral intention. According to the study, perceived usefulness, and flow experience influence attitudes significantly. A moderating effect of gender is observed on perceived ease of use on the path to behavioral intention, and the results extend the theoretical research on the use of metaverse technology for basketball instruction and TAM.

**Discussion:**

A metaverse-based learning experience can enhance the flow experience of basketball learning, thus increasing the willingness to use and the effectiveness of learning.

## 1. Introduction

In 2019, COVID-19 outbreak impacts the education system across more than 200 countries, affecting nearly 1.6 billion learners in more than 200 countries. Schools and education and training institutions around the world have been temporarily closed, affecting more than 94% of learners (Pokhrel and Chhetri, 2021; Karakose et al., 2022). COVID-19 outbreak has created a new norm in terms of crisis management approach, transformation of online learning, etc. Its outbreak has highlighted the importance of online learning worldwide (Adeoye et al., 2020). With its flexibility, convenience, space, and convenience, online learning has become an integral part of the educational process during the COVID-19 epidemic (Alqahtani and Rajkhan, 2020; Dhawan, 2020). While E-learning provides teachers and students with greater flexibility and comfort, it also has some disadvantages, such as low efficiency of learning (Roman and Plopeanu, 2021). As a new immersive solution for distance learning, Metaverse uses virtual reality as its key technology (Netland and Hines, 2021). Using virtual reality, higher education students can interact visually and digitally with complex concepts and dynamic objects, and it is possible to improve their learning outcomes under the premise of active learning (Fabris et al., 2019). The use of immersive VR in virtual simulation learning can enhance students’ experience of presence (Makransky et al., 2019). A mature virtual platform for education, Second Life is more suitable for Metaverse. It provides a supportive environment for facilitating classroom-style learning activities. It is capable of conducting experiments, task selection, creation, and dynamic feedback, which are conducive to improving students’ learning experiences (Jarmon et al., 2009; Warburton, 2009). Liu and Steed (2021) perception experiments were conducted using six multiplayer games on the Oculus Quest VR platform. Despite the fact that each platform presents challenges in terms of functional layout, working mechanisms, detail interaction, motion capture, etc., the task-based exercise proved to be effective. A number of studies regarding multimodal teaching have also demonstrated that the seamless interconnection of multichannel hardware and software can provide students with a high level of multisensory stimulation and enhance their learning experience (Tuo and Long, 2022). As metaverse technology and equipment continue to advance, new opportunities for online physical education have arisen. Using the metaverse to teach physical education will enable an improved online teaching environment, develop a more ideal online learning process, and enable students to more effectively acquire physical education knowledge and skills.

In the context of COVID-19, the quality and improvement requirements of online teaching systems are increasing, and schools and developers of online teaching systems are paying more attention to methods and systems. It appears that physical education teaching in Chinese colleges and universities is not utilizing technology effectively, and the traditional experiential teaching method is inefficient, thus restricting the teaching efficiency and the learning effect. The COVID-19 pandemic, however, has forced physical education classes to be conducted non-face-to-face. E-learning cannot meet the teaching requirements. Students cannot interact with each other. Teacher inspection efficiency is low, and tactical courses cannot be conducted (Gumantan et al., 2021). Metaverse is a multi-user immersive network environment capable of real-time, concrete, and dynamic interaction between users’ digital images (Mustafa, 2022). A new way of thinking has been brought about by the innovation of the metaverse concept. Is it possible to use metaverse technology as a tool to create an online teaching environment that meets the real-time interaction of online multi-persons, the sense of presence, the sense of action, the combined use of multisensory virtual reality, motion capture, and real-time feedback for online teaching of physical education courses reform is of great importance.

With online learning, not only can space and time limitations be broken, but also advanced teaching methods and high-quality educational resources can be incorporated, which has distinct advantages in terms of application (Kumar Basak et al., 2018). In addition, the continuous research and development of high-tech products, including 5G, augmented reality (AR), virtual reality (VR), and extended reality (XR) technology, has accelerated the realization of Metaverse technology (Wang et al., 2022). There has been an increase in metaverse research activity in recent years, and people’s interaction in the virtual space is becoming more direct and immediate. Combined with virtual reality, the technical characteristics of the metaverse technology are ideal for sporting events that require physical activity, interaction, social interaction, physical perception, and repetitive exercises (Juditya et al., 2021; Siahaan and Sawir, 2022). Especially in basketball courses, teachers and students interact more frequently, requiring the students to continuously familiarize themselves with the ball and practice technical and tactical cooperation on a regular basis. The existing online teaching method cannot provide high-frequency practice or interactive basketball lessons, so the combination of basketball courses with metaverse technology, coupled with the rapid development of technology, makes up for the shortcomings of online teaching, making basketball teaching methods more efficient and more diverse. This study combines metaverse technology in physical education with basketball courses to provide a reference for the application of metaverse technology in physical education. Although the current metaverse technology is still in its infancy, and its combination with sports has yet to produce good results, it provides a reference for the use of metaverse technology in physical education.

The COVID-19 virus was discovered more than 3 years ago, and its impacts have negatively impacted normal teaching in most countries (Zhou and Li, 2020; Lavidas et al., 2022). The teaching of physical education courses requires that students understand technical movements, tactics, and acquire these movements with guidance and demonstration by the teacher throughout the process, and the need for real-time interaction is high, especially in long-term repeated online courses. The metaverse technology can solve some of these disadvantages (Yang et al., 2022). The purpose of this paper is to discuss how metaverse technology can be applied in basketball courses by placing students in a virtual reality environment, allowing them to interact fully with their teachers, and enabling them to complete course goals more efficiently. The purpose of this paper is to discuss students’ intention to use new technologies, to improve students’ online and mixed classroom learning effects, and to provide references for the development and practical application of metaverse technology in the area of physical education.

### 1.1. Research purposes

As a first step, we combined metaverse technology with basketball teaching, discussed the application of metaverse technology for basketball teaching, and analyzed its feasibility and technical advantages. The influence of combining a technology acceptance model on college students’ willingness to utilize metaverse technology for basketball learning was discussed as well. Additionally, the new structural equation model is developed and suggestions are made for the teaching setting of metaverse technology basketball by analyzing the influence relationship and path coefficient among five variables: perceived usefulness, perceived ease of use, flow, attitude, and behavioral intention. Additionally, we examined whether gender moderates the path relationship. The purpose of this study is to identify the factors that affect college students’ willingness to use metaverse basketball, and to develop new directions for teaching and research in physical education. The findings are of wide-reaching significance because they highlight the significance of research and development on metaverse sports and how it affects the overall process.

## 2. Literature review

### 2.1. Applications and technologies related to the metaverse

“Snow Crash,” a science fiction novel published in 1992, introduced the concept of “Metaverse” for the first time (Joshua, 2017). With the release of the film “Ready Player One,” the virtual reality concept of OASIS band became more widely known to the general public. The immersive game enables players to escape from the real world through wearable VR devices, and through device operations, they can interact with other players in a virtual world that features an independent economic and social system to experience a variety of lifestyles(Nordstrom, 2016). Metaverse is a post-real world that integrates digital, virtual, and physical reality with multisensory interaction (Mystakidis, 2022). This is a new form of Internet application that combines blockchain technology, XR technology, high-speed wireless communications networks, digital twins, and other technologies. Metaverse is a blend of virtual reality and augmented reality, physical reality and digital virtuality, and a persistent multi-user environment. The tactile experience equipment through VR glasses can achieve a high sense of immersion and interactivity (Mystakidis, 2022). As a result of the meta-society running on the virtual Internet, people in different regions are able to interact and immerse themselves in a more realistic manner (Wang et al., 2022). In spite of the fact that the metaverse is more futuristic and powered by technologies like virtual reality, 5G, augmented reality, and artificial intelligence, the “big bang” of digital networks is not far away (Lee et al., 2021).

The development of metaverse technology is currently underway in the fields of social networking, language education, online conferences, manufacturing training, games, etc. (Siyaev and Jo, 2021a; Jovanović and Milosavljević, 2022). As AR, VR, and other technologies penetrate into the field of education, and metaverse wearable devices (such as Oculus, Ghost Pacer Glasses, Vive Flow, etc.), motion capture devices (such as VDSuit Full, etc.), cold and hot somatosensory devices (Pebble Fee), tactile sensing devices (Tactglove), and brain-computer interface (BCI) continue to advance (Egliston and Carter, 2022; Syvertsen, 2022). As technical integrity improves, it tends to be perfected. In a study by Kanematsu et al. (2014), virtual reality game platforms such as minecraft and roblox also provide educators with a platform for metaverse teaching (Rospigliosi, 2022). The use of e-learning systems brings more opportunities for educators to do scientific research and innovative teaching. In order for e-learning systems to be effective, they must be compatible with educational principles and theories (Katsaris and Vidakis, 2021).

### 2.2. Education and the metaverse

In the field of education, metaverse technology is of great significance. Although there is still no independent reference application, the development of related technologies and applications is taking shape, and it may have a significant impact in the future. With the development of good human-machine interaction and the realization of virtual reality, real-time interaction of virtual avatars has been made possible throughout many fields such as education and medicine. The use of augmented reality makes online interaction of real people more vivid and concrete (Genay et al., 2021; Aguayo et al., 2022). Metaverse has gradually evolved toward artificial intelligence in the field of educational technology, allowing students to experience a virtual classroom with real classroom elements, and this is more engaging than simple online learning (Tlili et al., 2022). The Lifelogging Metaverse integrates augmented reality with daily communication and is a useful tool for improving the ability to communicate in the classroom on a regular basis (Kye et al., 2021). Siyaev and Jo (2021b) use metaverse technology in blended learning of aircraft maintenance, voice interaction module, and blended interaction have significant effects on education and training. It is important to keep in mind that even if the existing technology is sufficient to support the metaverse, the expansion of economic and technological coverage will allow the metaverse to achieve its full potential (Wiederhold, 2022).

### 2.3. Instructing basketball

The use of virtual reality technology with basketball subjects makes online teaching more participatory and interactive, with all-round and multi-angle actions. Demonstration makes the teaching effect more effective (Shi, 2021). Unlike ordinary basketball courses taught in colleges and universities, basketball instruction is characterized by collectiveness, antagonism, fewer class hours, and greater content (Jiang et al., 2016). Teaching basketball involves teaching student’s basic basketball skills and refereeing knowledge through the interaction between educators and students (Aufan et al., 2019). Learning basketball skills and tactics is hindered by the language abstractions and limitations of traditional teaching methods (Chen and Wang, 2021). As a result of the system designed for the basketball class, college students were able to improve their skill proficiency and effectiveness of learning (Guo and Niu, 2021). The game training module in the teaching system has a very positive effect on improving the basic tactical coordination of students’ basketball skills (Lin and Liu, 2021). Online tests can effectively test the mastery of basketball rules by students through the online test module (Li and Li, 2021). Therefore, developing basketball teaching applications and digitizing resources has become an integral part of basketball education (Ma, 2021). It has been demonstrated that online basketball teaching improves student interest and teaching effectiveness, especially in situations where face-to-face instruction is not possible or distance restrictions apply, and its use will become more urgent as time progresses.

### 2.4. Technology acceptance model

As a model for explaining and predicting the acceptance of a specific type of technology (Yeou, 2016), TAM is one of the most commonly used Information Systems Acceptance (IS) models in the research on social media adoption and acceptance. It is primarily used to study the acceptance and use of social media by students and educators (Al-Qaysi et al., 2020). The rational behavior theory has become one of the most valuable models for predicting people’s acceptance or rejection of technology with the continuous development of rational behavior theory in psychology (Granić and Marangunić, 2019). As the most commonly used acceptance measurement model, TAM has the characteristics of adaptability, simplicity, and reliability (Al-Emran et al., 2018; Liao et al., 2018). This study illustrates the mutual influence relationship between the various facets of the massive open online course (MOOC) by using a modified technology acceptance model.

#### 2.4.1. Perceived ease of use

An individual’s perception of ease of use is the degree to which they think that using a particular system is effortless (Davis, 1989). Past experience indicates that perceived ease of use increases the willingness of users to use technology. When a customer perceives given software as easy to use, their willingness to use it will increase (Singh and Srivastava, 2018). Perceived ease of use also plays a significant role in human-computer interaction. A college student’s perception of computer ease of use is an important factor in promoting the use of e-learning by his or her peers (Ibrahim et al., 2017). It has been shown that perceived ease of use can have a positive impact on attitudes toward e-learning through behavioral intentions (Shao, 2020). In other words, if users perceive that the technology is easy to use and reduces their energy investment, it may also encourage their willingness to use it and their positive evaluation of it. However, in the new technological environment of the metaverse, it is worthwhile to consider whether perceived ease of use can still be considered important. Accordingly, we propose the following hypothesis:

*Hypothesis 1*: (H1) Perceived ease of use has a positive impact on college students' (H1a) behavioral intention and (H1b) attitude in using metaverse technology for learning basketball.

#### 2.4.2. Perceived usefulness

Originally, perceived usefulness was defined as the extent to which a person believed that using a particular system would enhance their efficiency in the workplace (Davis, 1989). Users’ willingness to use a product is influenced by their perception of its usefulness. In the case of US residents, perceived usefulness plays a significant role in their willingness to use smart technology (Chen et al., 2017). Participants are more likely to accept and use a given system service when they believe that it will benefit them in some way (Kamal et al., 2020). Thus, it is one of the deciding factors for users to accept and use the system. Similarly, pre-service English teachers’ perceptions of the usefulness of using m-LMS in classroom instruction have a significant effect on their attitudes toward this technology (Mukminin et al., 2020). Users’ attitudes and behavior may be influenced by useful perception, which promotes the formation of positive cognition. Based on these findings, we propose the following hypotheses regarding the relationship between perceived usefulness and behavioral intention and attitude:

*Hypothesis 2*: (H2) Perceived usefulness has a positive impact on college students' (H2a) behavioral intention and (H2b) attitude in using metaverse technology for learning basketball.

#### 2.4.3. Attitude

Research in psychology focuses on attitudes. A change in attitude (AT) will change one’s behavior and will (Howe and Krosnick, 2017). A person’s attitude can be used to reflect not only their preferences, but also their evaluations of a phenomenon. In the previous study of the attitude of students in virtual environments, its importance was noted. An important factor influencing a student’s willingness to utilize e-learning is his or her attitude toward the technology (Mailizar et al., 2021). Behavioral intentions when using technology are also affected by attitudes (Mohd Amir et al., 2020). It has been noted by Rad et al. (2022) that a good attitude and a willingness to use technology are important influencing factors for technology adoption (Rad et al., 2022). The interaction between students’ attitudes and teachers’ participation in a blended learning environment allows students to experience the benefits of e-learning, which, in turn, increases their willingness to use and their behavior toward it (Keržič et al., 2019). Behavioral intentions are determined by attitudes toward using the system and perceived utility, while attitudes are determined by perceived utility and perceived ease of use (Ramírez-Correa et al., 2019). This means that positive attitudes are behavioral intention triggers, which are positively correlated with behavioral intentions and have a positive impact on the perception of behavioral control. We propose the following hypotheses in order to verify the relationship between attitude and behavioral intention and usage behavior in this study:

*Hypothesis 3*: (H3) Attitude has a positive impact on college students' (H3a) behavioral intention and (H3b) use behavior in using metaverse technology for learning basketball.

#### 2.4.4. Behavioral intention

Behavioral intention (BI) can be defined as the intention to consciously decide whether to engage in a particular behavior in the future (Ramírez-Correa et al., 2019). In online education, a student’s behavioral intention refers to his or her intention to use a particular technology or application. Basically, it is the tendency of a person to perform certain behaviors (Fishbein and Ajzen, 1977). Studies on TAM and education have predicted the relevant variables in the model in the past. An individual’s intention to use a system is positively influenced by perceptions of the system’s ease of use and usefulness, and a high level of willingness to use improves the user’s behavior in utilizing the system (To et al., 2021). According to the TAM model, the use behavior of college students toward E-Learning is significantly influenced by their intention to use (Mohamad et al., 2019). It is the behavioral tendency that determines the final participation performance of behavior (Ajzen and Processes, 1991). We investigate the relationship between students’ behavioral intentions and their use of metaverse technology in the teaching of basketball in this article. Our hypothesis is therefore as follows:

*Hypothesis 4*: (H4) Behavioral intention has a positive impact on college students' (H4a) use behavior in using metaverse technology for learning basketball.

### 2.5. The flow theory

As defined by Csikszentmihalyi, flow occurs when people are engaged in an activity and lose track of time and awareness of their surroundings (Csikzentimihalyi, 1975). A state of flow is when one is completely absorbed in what they are doing, losing all sense of time and space (Oliveira dos Santos et al., 2018). Human-computer interaction education can lead to students showing higher levels of concentration when they are in a state of flow. The results of pioneering research indicate that online flow reduces participants’ self-awareness, increases their engagement with the site and content, and allows them to focus on the experience they are having (Ampong et al., 2018). There will be a reduction in anxiety, as well as excitement and fulfillment associated with the flow state. Flow experiences at work can improve participants’ well-being and engagement (Ilies et al., 2017). By adding serious games to learning, flow experience can promote the learning effect, which contributes to achieving the expected learning effect and can be used to assess serious game quality (Perttula et al., 2017). In the event that users experience the positive effects of being in a flow state, their behavior intentions and attitudes are likely to change for the better. Our study uses the virtual world and game attributes of the metaverse as the background, so it is more interesting in and of itself, and we investigate the possible influence of flow on attitude and behavioral intentions. Accordingly, we propose the following hypothesis:

*Hypothesis 5*: The (H5) flow experience has a positive impact on college students' (H5a) behavioral intention and (H5b) attitude in using metaverse technology for learning basketball.

### 2.6. Gender moderator

Previous educational studies have used gender as a key moderating factor. The impact of perceived ease of use on behavioral intentions will be moderated by gender, and males will show greater significance in the use of technology (Lwoga and Lwoga, 2017). There is also a basic element to technology that targets user differences, which may also involve the types of needs of different users. When these needs are high, a certain attention may be given to the ease of use of existing and new technology. According to Song et al. (2021b), perceived ease of use is an important predictor of attitude toward APPs, and gender appears to moderate this effect. The ease of use of APPs may be related to the fact that men are usually more interested in exploring new things, while women tend to be more interested in their existing experience tendencies (Ventre and Kolbe, 2020). There may be differences between perceptions of usefulness and attitudes and willingness in some specific task environments as a result of the benefits that different genders place a greater emphasis on. According to the study of Keržič et al. (2019), gender is also a significant moderator of attitude on behavioral intention and use of behavioral paths in blended learning. There is, however, no correlation between gender and students’ willingness to use Zoom when it is actually used (Alfadda and Mahdi, 2021). Gender differences in contemporary society are, in fact, not very significant in many areas, such as cognition and experience. Gender has little impact on the path of flow to attitude or behavior intention in many studies (Sakkthivel and Ramu, 2018; Mahfouz et al., 2020). However, gender differences are not reflected in all tasks. In order to test the effect of gender on each path of the model, we propose the following hypothesis:

*Hypothesis 6*: There is a moderating effect of (H6) gender on (H6a) perceived ease of use on attitude; (H6b) perceived ease of use on behavioral intention; (H6c) perceived usefulness on attitude; (H6d) perceived usefulness on behavioral intention; (H6e) attitude on behavioral intention; (H6f) attitude on use behavior; (H6g) behavioral intention on use behavior; (H6h) flow on behavioral intention; (H6i) flow on attitude; when college students use metaverse technology to learn basketball.

## 3. Materials and methods

In this study, we surveyed respondents’ opinions online and obtained their consent without interacting with them or encountering medical ethical concerns. Due to this, there is no statement applicable to the institutional review board.

### 3.1. The survey object

The purpose of this study is to explore the influencing factors of college students’ willingness to use metaverse technology in basketball teaching. A structural equation model is used to examine the relationship between various factors. An introduction to the potential form of participation in metaverse basketball in the future is contained in the questionnaire, which includes both a video of the metaverse concept as well as the current metaverse resource video related to basketball. Using freshmen and sophomores in college who have basketball courses as the research object, this paper explores factors that may influence students’ willingness to use metaverse technology in the teaching of basketball courses.

A basic understanding of metaverse-related products and technical knowledge ensures that college students can make more objective evaluations. Additionally, college basketball courses are only offered in the first and second grades, thus respondents’ learning needs are higher for basketball courses than for other levels. Further, as a result of the objective factors of COVID-19, Chinese college students have gained extensive experience in learning online courses in recent years, thus facilitating a better comparison when they join the metaverse basketball education program. Therefore, we have chosen them as the subject of our research.

### 3.2. Design of questionnaires

We conducted a convenience sampling survey online from February to March 2022 using a five-point Likert scale ranging from 1 for strongly disagree to 5 for strongly agree. The questionnaire description was made available through the questionnaire link, and those participating in the survey did so voluntarily and with full knowledge of the survey.

On the basis of the original TAM questionnaire, important factors relevant to students using metaverse technology for basketball learning are supplemented. The heart flow experience has been modified for related questionnaires. The latent variables refer to sources, codes, items, and source information (Table 1).

**Table 1:** The scale of measurement.

Latent variable	Coding	Item	Source
Behavioral intention	BI1	In the future, I intend to continue using metaverse technology for basketball learning.	Lee and Lee (2020)
	BI2	My daily life will always involve using metaverse technology for the purpose of learning basketball.	
	BI3	It is my intention to continue using metaverse technology for basketball studies on a regular basis.	
Use behavior	AC1	It is my pleasure to use metaverse technology to learn basketball.	
	AC2	In order to learn about basketball, I will actively utilize metaverse technology.	
	AC3	For basketball learning, I would recommend metaverse technology to anyone around me.	
	AC4	As a basketball learner, I am confident in the use of metaverse technology.	
Perceived ease of use	PEU1	It is easy for me to learn the metaverse technology for basketball learning.	Lam et al. (2020)
	PEU2	For me, the metaverse technology is easy to navigate and find the information I need for basketball learning.	
	PEU3	I have found that metaverse techniques for basketball learning are easy to master.	
	PEU4	Metaverse technology makes teaching basketball easy for me.	
Perceived usefulness	PU1	In my studies of basketball, I find the metaverse technology to be extremely helpful.	Lam et al. (2020)
	PU2	My studies in basketball have been enhanced by the use of metaverse technology.	
	PU3	My basketball studies are facilitated by the use of metaverse technology.	
	PU4	The metaverse technology could provide me with the information I need to learn basketball.	
Attitude	AT1	If I were given the opportunity to utilize the metaverse for basketball learning, I would have a favorable opinion of it.	Jiang et al. (2022)
	AT2	The metaverse technology provides valuable services for basketball learning.	
	AT3	Using metaverse technology to learn basketball can be an enjoyable experience.	
Flow	F1	Learning to play basketball using metaverse technology may cause me to lose focus on what is going on around me.	Chen et al. (2018)
	F2	With the use of metaverse technology, I expect I will lose my sense of time and feel like time flies when I learn basketball.	
	F3	When I learn basketball through the use of metaverse technology, I anticipate being very focused.	

### 3.3. Collection of data

During this study, 1,317 samples were collected. After removing 478 invalid samples, there were 839 remaining samples, and the effective recovery rate was 63.71%. Based on valid samples, data statistics are performed, and the results are presented in Table 2. Since Chinese college students take general basketball physical education courses as freshmen and sophomores, this study selected 292 boys and 547 girls who are freshmen and sophomores who are interested in basketball. Specifically, the sample population was drawn from the eastern, central, western, and northern regions, as well as students specializing in the natural sciences, medicine, social sciences, etc. For the purpose of this study, the content is representative of these regions.

**Table 2:** Basic information of interviewees.

Sample	Category	Number	Percentage
Gender	Male	292	34.8
	Female	547	65.2
Grade	Freshman	530	63.2
	Sophomore	309	36.8
Major	Natural science	89	10.6
	Engineering and Technology	191	22.8
	Medicine and health Sciences	125	14.9
	Agricultural Science	45	5.4
	social sciences	172	20.5
	Humanities	125	14.9
	Science of physical culture and sports	92	11
Hometown	Eastern Region	218	26
	Middle Region	336	40
	Western Region	254	30.3
	Northeast Region	30	3.6
	Hong Kong, Macao, and Taiwan regions	1	0.1

A chi-square test was conducted on the data collected in the three stages, as shown in Table 3. There were no significant differences between the groups in the results (*p* > 0.05). There is therefore homogeneity in the responses to each of the basic information questions. The results of the analysis of the recovered samples should therefore be extrapolated to the parent population.

**Table 3:** Testing for non-response bias.

	χ^2^	DF	*p* value
Gender	1.265	2	0.531
Grade	2.486	2	0.288
Hometown	13.216	8	0.105

## 4. Results

### 4.1. Descriptive statistics

Due to the pre-assumptions made in the statistical method used in this study for analyzing the recovery data, the corresponding hypothesis test is performed following data collection, as shown in Table 4. It is evident from the skewness and kurtosis of the data for each dimension in this study, as well as the normality of the test distribution, that the absolute value of skewness for each dimension data is between 0.744 (CI) and 0.611(FE). Kurtosis lies between 0.923 (CH) and 0.416 (FE). Accordingly, the total skewness of the data collected by the questionnaire test is less than 3.0, and the total kurtosis is less than 8.0, indicating univariate normality (Kline, 2015).

**Table 4:** Descriptive statistical results.

Constructs	Mean	S.D.	Skewness	Kurtosis
FL	3.608	0.760	−0.611	0.466
PEU	3.611	0.724	−0.668	0.749
PU	3.692	0.721	−0.694	0.784
AT	3.702	0.728	−0.699	0.845
BI	3.590	0.805	−0.672	0.416
UB	3.660	0.743	−0.744	0.923

### 4.2. Analyses of reliability

The questionnaire is verified using Cronbach’s Alpha and corrected total correlation coefficient (CITC). As shown in Table 5, the CITC of all facets is greater than 0.5 and the Cronbach’s Alpha reliability coefficient is greater than 0.7. Consequently, the questionnaires and scales used in this study have a high level of internal consistency.

**Table 5:** Analyses of reliability.

Item	Corrected item total correlation	Cronbach’s alpha if item deleted	Cronbach’s alpha	Item	Corrected item total correlation	Cronbach’s alpha if item deleted	Cronbach’s alpha
FL1	0.599	0.739	0.783	AT1	0.697	0.754	0.829
FL2	0.654	0.674		AT2	0.698	0.754	
FL3	0.620	0.710		AT3	0.668	0.783	
PEU1	0.736	0.823	0.867	BI1	0.737	0.799	0.859
PEU2	0.730	0.826		BI2	0.743	0.794	
PEU3	0.703	0.837		BI3	0.721	0.814	
PEU4	0.707	0.835		UB1	0.760	0.825	0.873
PU1	0.741	0.833	0.873	UB2	0.725	0.840	
PU2	0.729	0.838		UB3	0.722	0.841	
PU3	0.725	0.839		UB4	0.707	0.846	
PU4	0.717	0.842					

### 4.3. Analyses of exploratory factors

According to Table 6, when exploratory factor analysis is used to verify the individual facets of each facet, all constructs have Kaiser-Meyer-Olkin (KMO) values greater than 0.7, and the significance of the Bartlett sphericity test is less than 0.05. It is therefore possible to conduct an exploratory factor analysis (Norusis, 1992). Each construct item has a commonality greater than 0.5, indicating a high degree of overlap (Hair et al., 1998). There is only one new factor more than 0.6 that can be extracted with an eigenvalue greater than 1, and the total explained variation is greater than 60%, indicating that it follows single-dimensionality and is suitable for further analysis.

**Table 6:** Results of the exploratory factor analysis.

Construct	KMO	Bartlett’s Sphere Test	Item	Commonality	Factor loading	Eigenvalue	Total variation explained
FL	0.701	0.000	FL1	0.671	0.819	2.103	70.101%
			FL2	0.733	0.856		
			FL3	0.699	0.836		
PEU	0.833	0.000	PEU1	0.736	0.858	2.864	71.601%
			PEU2	0.729	0.854		
			PEU3	0.697	0.835		
			PEU4	0.702	0.838		
PU	0.834	0.000	PU1	0.740	0.860	2.898	72.458%
			PU2	0.726	0.852		
			PU3	0.721	0.849		
			PU4	0.712	0.844		
AT	0.722	0.000	AT1	0.756	0.870	2.238	74.593%
			AT2	0.757	0.870		
			AT3	0.725	0.852		
BI	0.735	0.000	BI1	0.784	0.885	2.341	78.025%
			BI2	0.790	0.889		
			BI3	0.767	0.876		
UB	0.834	0.000	UB1	0.762	0.873	2.899	72.486%
			UB2	0.721	0.849		
			UB3	0.717	0.847		
			UB4	0.700	0.837		

### 4.4. Analyses of confirmatory factors

#### 4.4.1. Validation of convergent validity

This study used confirmatory factor analysis (Confirmatory Factor Analysis) to analyze the data. In this study, AMOS 22.0 was selected for analysis. Table 7 illustrates that the standardized factor loading is greater than 0.62 (Wei et al., 2022). Squared multiple correlation (SMC) is greater than 0.4, *t* value is greater than 2.58, *p* value is significant, combination reliability is greater than 0.7, and the average variance extraction (AVE) is greater than 0.5. Thus, this structure has good convergent validity (Fornell and Larcker, 1981).

**Table 7:** Validation of convergent validity.

	Items	Factor loading	*t* value	*p* value	SMC	AVE	CR
FL	FL1	0.644	19.232	0.001	0.414	0.547	0.782
	FL2	0.749	23.356	0.001	0.561		
	FL3	0.816	26.065	0.001	0.665		
PEU	PEU1	0.795	26.692	0.001	0.632	0.621	0.868
	PEU2	0.803	27.104	0.001	0.645		
	PEU3	0.775	25.721	0.001	0.601		
	PEU4	0.780	25.966	0.001	0.609		
PU	PU1	0.806	27.285	0.001	0.650	0.632	0.873
	PU2	0.800	26.988	0.001	0.641		
	PU3	0.789	26.416	0.001	0.622		
	PU4	0.786	26.295	0.001	0.618		
AT	AT1	0.780	25.481	0.001	0.608	0.620	0.830
	AT2	0.804	26.602	0.001	0.646		
	AT3	0.777	25.372	0.001	0.604		
BI	BI1	0.822	27.814	0.001	0.676	0.670	0.859
	BI2	0.830	28.225	0.001	0.689		
	BI3	0.804	26.932	0.001	0.647		
UB	UB1	0.823	28.176	0.001	0.678	0.634	0.874
	UB2	0.798	26.879	0.001	0.636		
	UB3	0.784	26.218	0.001	0.615		
	UB4	0.779	25.965	0.001	0.607		

According to Table 8, the model fitting indicators are in line with the recommended indicators, indicating a good fitting effect (Baik et al., 2020). There is a better model fit for the first-order CFA model. In addition to measuring common method bias, the data were also tested using the common latent factor method (CCLFM). It appears that CCLFM’s model fitting results are not significantly superior to those of CFA. CCLFM’s RMSEA and SRMR values decreased by less than 0.05 compared with the CFA model, and GFI, AGFI, NFI, and CFI’s values decreased by less than 0.1. Therefore, this study does not have a common method bias problem.

**Table 8:** Model fitting index comparison results of CFA and CCLFM.

Common indices	χ^2^/df	RMSEA	GFI	IFI	CFI	TLI	SRMR
Judgment criteria	<3	<0.08	>0.9	>0.9	>0.9	>0.9	<0.08
JCFA Value	2.227	0.038	0.958	0.980	0.980	0.976	0.026
CCLFM Value	1.897	0.033	0.964	0.986	0.986	0.982	0.021

#### 4.4.2. Validation of discriminant validity

Data discriminant validity was determined using the Fornell-Larcker criterion method in this study. Table 9 indicates that each facet’s square root of AVE is greater than its correlation coefficient with other structures, indicating good discriminant validity for each construct (Fornell and Larcker, 1981). Additionally, the Heterotrait-Monotrait Ratio (HTMT) tests showed an improvement in the discrimination ability of each dimension. According to previous studies, the HTMT values for each dimension were less than 0.85, which is within the range recommended (Peralta and Rubalcaba, 2021). As shown in Table 10, the dimensions measured in this study have good discriminant validity.

**Table 9:** Test of discriminant validity.

	FL	PEU	PU	AT	BI	UB
FL	0.740					
PEU	0.561	0.788				
PU	0.529	0.672	0.795			
AT	0.539	0.621	0.673	0.787		
BI	0.531	0.647	0.578	0.585	0.819	
UB	0.490	0.672	0.660	0.622	0.676	0.796

**Table 10:** Results of Heterotrait-monotrait ratio.

	FL	PEU	PU	AT	BI	UB
FL	/					
PEU	0.686	/				
PU	0.683	0.771	/			
AT	0.813	0.732	0.791	/		
BI	0.783	0.749	0.667	0.844	/	
UB	0.599	0.772	0.756	0.731	0.780	/

### 4.5. Results of structural equation modeling

**Figure 1:**
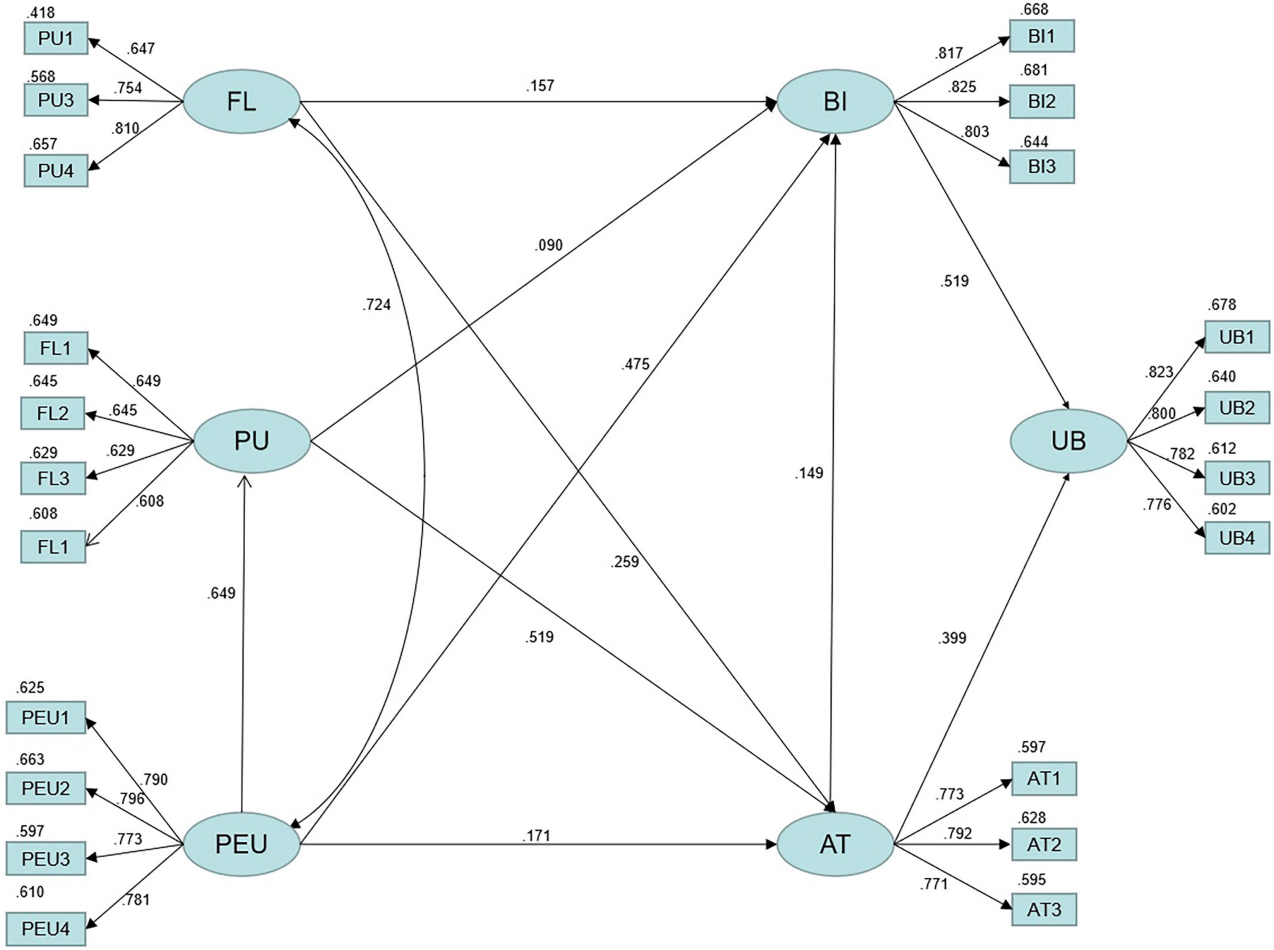
Structural equation model.

Figure 1 shows the model path relationship. Table 11 indicates that the model fitting indicators are all higher than the recommended standard values. Additionally, the model has a good fitting index, indicating that it is reasonably constructed and has reached the standard level of fit (Fornell and Larcker, 1981).

**Table 11:** Adaptability of SEM.

Common indices	χ^2^	df	χ^2^/df	RMSEA	GFI	IFI	CFI	TLI	SRMR
Judgment criteria			<3	<0.08	>0.9	>0.9	>0.9	>0.9	<0.08
Value	467.345	178	2.626	0.044	0.950	0.973	0.973	0.968	0.034

This paper employs the maximum probability similarity method to estimate the direct and indirect effects of each dimension path. According to Table 12, PEU significantly predicts PU (ß = 0.789, *p* = 0.001). BI is directly affected by FL (ß = 0.196, *p* = 0.026), and PEU (ß = 0.632, *p* = 0.001). The study indicates that PU, FL, and PEU have a positive causal relationship with the BI of college students who adopt metaverse technology to learn basketball. At the same time, PU (ß = 0.167, *p* = 0.097) and AT (ß = 0.149, *p* = 0.237) failed to establish a significant route relationship with BI, indicating that AT has no effect on the BI of college students who use metaverse technology to learn basketball.

**Table 12:** Direct and indirect effects.

Path	Direct effect		Indirect effect		Total effect	
	β	B-C Sig.	β	B-C Sig.	β	B-S Sig.
PU → BI	0.090	0.462	0.077	0.197	0.167	0.097
PU → AT	0.519	0.001	/	/	0.519	0.001
FL → BI	0.157	0.083	0.038	0.135	0.196	0.026
FL → AT	0.259	0.005	/	/	0.259	0.005
PEU → PU	0.789	0.001	/	/	0.789	0.001
PEU → BI	0.475	0.001	0.158	0.038	0.632	0.001
PEU → AT	0.171	0.183	0.409	0.001	0.580	0.001
AT→BI	0.149	0.237	/	/	0.149	0.237
PU → UB	/	/	0.294	0.001	0.294	0.001
PEU → UB	/	/	0.560	0.001	0.560	0.001
FL → UB	/	/	0.205	0.001	0.205	0.001
AT→UB	0.399	0.001	0.077	0.212	0.476	0.001
BI→UB	0.519	0.001	/	/	0.519	0.001

Technology acceptance model factor and FL significantly affected UB. PU indirect effect (ß = 0.294, *p* = 0.001), PEU indirect effect (ß = 0.560, *p* = 0.001), FL indirect effect (ß = 0.205, *p* = 0.001), AT direct effect (ß = 0.476, *p* = 0.001), BI direct effect (ß = 0.519, *p* = 0.001). It is shown that PU, PEU, FL, AT, and BI are associated with the UB of college students who use metaverse technology in order to learn basketball.

Perceived ease of use (ß = 0.560, *p* = 0.001) and PU (ß = 0.519, *p* = 0.001) directly impact AT. There is a direct and significant predictive effect of FL (ß = 0.259, *p* = 0.005). There is evidence that PEU, PU, and FL have a positive impact on the AT of college students using metaverse technology to learn basketball.

Furthermore, this paper adds a new competition model to each path relationship and establishes the null hypothesis (specify male β = female β model). Table 13 compares the competition model with the original model to examine the moderating effect of gender. It has been found that gender significantly modifies the path relationship between perceived ease of use and behavioral intentions in both the competition model and the original model. On the other paths, there is no significant adjustment effect. Our findings indicate that girls are more sensitive to perceived ease of use than boys. In general, female users prefer simple interaction modes in order to be willing to use it. They are more likely to adopt a positive attitude and be willing to accept technology when they perceive its use to be less challenging. Designing or teaching should therefore take into consideration the perceived needs of female users for ease of use.

**Table 13:** Gender moderating effect results.

Path	Original model				Specify male β = female β model				Model comparison	
	Male		Female		Male		Female		CMIN	*p*
	*β*	*p*	*β*	*p*	*β*	*p*	*β*	*p*		
PU → BI	0.099	0.595	0.064	0.683	0.083	0.581	0.073	0.586	0.029	0.865
PU → AT	0.525	0.006	0.501	0.001	0.532	0.001	0.497	0.001	0.012	0.913
FL → BI	0.235	0.116	0.072	0.527	0.161	0.134	0.148	0.126	1.935	0.164
FL → AT	0.204	0.134	0.313	0.018	0.262	0.009	0.267	0.008	1.238	0.266
PEU → PU	0.798	0.001	0.785	0.002	0.793	0.001	0.787	0.002	0.210	0.647
PEU → BI	0.291	0.159	0.601	0.003	0.481	0.004	0.472	0.003	6.372	0.012
PEU → AT	0.222	0.199	0.135	0.426	0.175	0.166	0.167	0.159	0.363	0.547
AT→BI	0.317	0.211	0.085	0.642	0.184	0.290	0.170	0.286	2.244	0.134
AT→UB	0.426	0.104	0.391	0.001	0.402	0.001	0.397	0.001	0.097	0.756
BI→UB	0.488	0.114	0.527	0.001	0.495	0.001	0.525	0.002	0.007	0.933

## 5. Discussion

The purpose of this paper is to investigate the influence factors of TAM model expansion on students’ willingness to use metaverse technology in basketball instruction. We conducted a correlation analysis on PU, PEU, FL, AT, BI, and UB to evaluate the moderating effect of gender on these variables. Using gender as a moderator variable to examine the relationship between college students’ willingness to learn basketball through metaverse technology. A significant aspect of this study is that the results present new findings based on existing literature and serve as a reference for the integration of metaverse technology in the field of college basketball education.

There is a positive causal relationship between PEU and BI for college students who use metaverse technology to learn basketball, especially among females. A system that is easy to use will give students a better experience, which will increase their willingness to use the new system (Song et al., 2021a). Google Classroom’s perceived ease of use is an important construct for predicting students’ willingness to use the program (Albashtawi and Al Bataineh, 2020). It is easy for them to obtain a comfortable experience as a result of the convenient operability. New instruments are not always easily accepted when used for the first time, but good ease of use during use increases learners’ willingness to use them in the future (Sugandini et al., 2018). This may be due to the fact that the metaverse technology is easier to operate after use, and it is more conducive to learning basketball skills and tactics, thereby generating positive behavioral intentions.

Despite the absence of a positive causal relationship between PEU and AT, we cannot rule out that some users’ adverse reactions to a variety of metaverse products or experience with VR glasses may affect their attitudes toward using metaverse technology in the future, which needs to be explored further in future studies (Gao et al., 2021). This non-significant correlation was also confirmed in PT Tokopedia’s user’s study of purchase intention (Gunawan et al., 2019). In contrast to our findings, some studies have demonstrated that perceived ease of use also affects users’ attitudes, and they believe that adding personalized instructions and e-guides will help neutralize users’ negative perceptions of technical complexity (Weng et al., 2018; Cheah et al., 2022). It may be that today’s college students are generally accepting of electronic products, and that new technologies and products, such as smart phones and virtual reality, are rarely difficult to use. Furthermore, it confirmed their desire to make use of cloud universe technology in the classroom. Considering the pioneering technology and equipment, designers should provide a convenient operating system that is in line with the developer’s past development experience based on the technology acceptance basis. However, teachers can also experiment with combining high-tech with teaching, which can better stimulate students’ enthusiasm for learning.

Perceived usefulness and BI did not have a positive causal relationship. The reason for this may be that college students lack sufficient knowledge and experience in terms of practicality since they are experiencing virtual teaching for the first time in their physical education courses. Unlike our results, previous studies have found that usefulness is often a significant factor affecting behavior. There is a significant correlation between perceived usefulness and willingness to adopt Web 2.0 technologies in education (Kazoka and Mwantimwa, 2019). Utilization of new educational technology is directly and positively influenced by its usefulness and ease of use (Moslehpour et al., 2018; Mutahar et al., 2018). Additionally, Santhanamery and Ramayah (2018) found that perceived usefulness is an important predictor of users’ intentions to use electronic filing systems.

Perceived usefulness and AT have a positive causal relationship. Students develop positive coping attitudes when they perceive that teacher feedback and diagnosis are useful in a formative assessment intervention system (Rakoczy et al., 2019). There is a qualitative relationship between perceived usefulness and attitudes toward blended learning (Nadlifatin et al., 2020). A teacher’s attitude toward mobile learning is directly affected by the perceived usefulness of the technology (Kalogiannakis and Papadakis, 2019). In college, there are often high expectations for new technologies. College students tend to be early adopters of new technologies. Because they believe it is useful, when they use the highly useful metaverse technology, they will exhibit a positive attitude, thereby demonstrating a higher level of commitment and willingness to use it.

In order to meet the special needs of physical education students, traditional face-to-face instruction is a necessary component of most physical education courses (Zhang et al., 2016). With the emergence of metaverse technology, face-to-face instruction can be enhanced with a new form of virtual reality. It is also important for physical education teachers to adjust their focus in order to stimulate students’ interest, not only through the use of teaching methods, but also by exploring new technology models in order to discover new system models that are appropriate for teaching physical education.

Attitude and BI did not show a positive causal relationship. Similar to the previous research, this study found that attitudes do not directly influence users’ willingness to use websites in the context of COVID-19 (Rachmawati et al., 2020). Contrary to our findings, Udayana et al. found that users’ usage attitudes positively influenced their behavior intentions (Udayana et al., 2022). A positive causal relationship exists between teachers’ attitudes toward multimedia and their intention to use it (Weng et al., 2018). It is important to note that users’ attitudes toward a certain product may directly affect their willingness to use it, but it is also influenced by many other factors such as usefulness, advertising effects, user evaluations, etc., but often a bad attitude formed after an experience is very difficult to change.

Attitude and UB were found to have a positive causal relationship, supporting previous research. In the midst of the COVID-19 pandemic, students expressed a positive attitude toward Zoom for online learning (Mohd Amir et al., 2020). Attitudes are also directly related to the use of new technologies (Upadhyay et al., 2018). When people adopt positive attitudes toward technology cognition, they are more likely to use technology, which, in turn, encourages them to continue using it. Attitudes toward products have a significant impact on their use and subsequent behavior (Um, 2019). The positive attitude of college students toward the use of Metaverse technology can encourage them to improve their grades and sports goals, thus displaying a positive attitude toward technology use. Users’ perceptions of the usefulness of this technology in basketball teaching applications should be taken into account by designers, in order to increase users’ willingness to use the technology and increase its use. It is also imperative for teachers to actively recommend and discover the value of technology to their students in order to communicate the usefulness of technology to them and improve their attitudes toward its use.

A positive causal relationship exists between BI and UI, and users’ intention to use APPs is positively affected by their usefulness and ease of use, which increases their usage behavior (Scheper et al., 2019). College students’ use of Metaverse technology will be influenced by their willingness to use (Kondrat, 2017). Metaverse technology will be used more actively by college students with positive behavioral intentions.

Flow theory and BI have a positive causal relationship. According to previous studies, students’ flow experiences with streaming devices significantly influence their intentions to use the device (Yang and Lee, 2018). It has been demonstrated that the generation of flow experiences has a significant impact on improving learning efficiency (Jeon et al., 2018). In contrast to our findings, some studies only found an indirect relationship between flow and behavior (García-Jurado et al., 2018; Kim et al., 2020). Using metaverse for basketball learning may stimulate students’ flow experience and positive behavior intentions due to its technical characteristics and virtual reality effects.

Flow theory and AT are positively related. In part, this may be due to the new features of the metaverse, which improve its appeal to students and arouse their curiosity. Most of the content is in the form of video games, which makes creating a flow experience easier, as a result of which the attitude in the classroom has become more positive (Arghashi and Yuksel, 2022). The flow experience using the AR program is believed to promote the formation of positive attitudes toward the brand. A student’s attitude to using the Arduino micro-development version of the teaching tool is directly influenced by their experience with flow (Zeeman and Botes, 2020). By utilizing VR technology, students are able to experience a smooth experience which leads to a change in attitude (Boroumand et al., 2021). The metaverse technology facilitates a higher level of recognition by students and a higher level of physical and mental involvement. For educational products, the flow experience will definitely be beneficial to the learning process. As part of the educational process, teachers should also integrate more high-end technologies; provide students with a variety of learning interaction modes, and make active recommendations to further enhance the learning experience.

The influence of gender on PEU on the behavioral intention path can be moderated. It was found that the same results were observed in studies examining users’ willingness to use the adoption of driverless vehicles (Koul and Eydgahi, 2018; Assaker, 2020). Contrary to our findings in articles concerning the acceptance of new economic technologies and smart watch adoption intentions (Liu and Yang, 2018; Dutot et al., 2019). We found that women value the convenience of technology and product operation more than men, and this will influence their willingness to utilize metaverse technology. The differences in interests between women and men make most girls have little practical experience operating VR, AR, and other equipment, so they pay more attention to the ease of use of new systems than boys do. When metaverse technology is used for teaching, the insignificance of gender’s moderating effect on other pathways indicates that there is no need to teach separately by gender. Designers do not need to set content differently according to gender for teaching purposes.

## 6. Contribution and suggestions

### 6.1. Theoretical contribution

The purpose of this study is to examine the relational path hypothesis and test whether college students are willing to engage in online basketball learning through metaverse technology. Studying how college students will use metaverse technology to learn basketball in order to determine the university’s acceptance of new technology. A significant contribution of this paper is to extend the theoretical research on the use of metaverse technology in basketball teaching, which will be useful for future research into the application of metaverse technology in teaching and scientific research. In addition to verifying the applicability of TAM in the teaching of basketball in the metaverse, this study expands the TAM model by adding flow experience and gender as a moderating factor. An important research direction is the expansion of TAM theory (Jiang et al., 2021). The positive effects of flow experiences have also been thoroughly explored in previous research in technology acceptance and education (Jackman et al., 2021). This study has confirmed that the integration of flow and gender into the TAM improved explanation of the application of metaverse technology in basketball teaching. The extended model can be used in the research of new technologies and equipment in the teaching field, as well as provide a reference for other scholars.

### 6.2. Significance for management

In the metaverse, the virtual environment provides the ultimate immersive experience, and the user interacts with virtual characters in a virtual environment. The content of online basketball instruction is rich and easy to understand when combined with metaverse technology, which improves the scope of knowledge acquisition and makes students’ basketball learning more convenient and systematic (Jin and Zou, 2021). Teachers in the metaverse environment have a greater variety of online basketball teaching opportunities and are more in touch with reality. As a result, teachers are able to provide students with error-correcting guidance and provide more targeted communication. Furthermore, they are able to pay attention to the needs and deficiencies of each student. Rich and colorful teaching methods, scenes, and interactive modes are used to stimulate student interest in learning and achieve better learning outcomes.

This study has the following practical contributions.

The results demonstrate that perceived ease of use is the most important factor affecting college students’ use of metaverse technology for basketball learning. Compared with general online education, when metaverse technology is used in basketball instruction, the new technology provides convenience in basketball learning, the technology is easier to use, and the learning tasks can be accomplished more efficiently, which has a profound effect on the learning effect of online basketball courses. The flow experience is another key construct that influences college students to use metaverse technology for basketball learning. As college students are enthusiastic about basketball and pay close attention to high-tech products, they are attracted to it in the process of using metaverse technology and stimulate their interest in exploration, which helps to enhance basketball in silent learning. It facilitates discovery of the technical advantages of the metaverse and can be applied to other fields or course learning as well. Additionally, perceived usefulness plays an important role. College students are the leaders of the future era, playing a pivotal role in the exploration and development of new technology. The use of metaverse technology is perceived to be more useful for online basketball learning, and students are more willing to use it for basketball training and training courses. This will not only accelerate the diversification of teaching methods, but will also stimulate students’ interest in exploring the metaverse.

## 7. Conclusion

As metaverse theory and technology continue to expand, we anticipate a breakthrough in virtual interactive body participation in the near future. It provides a prediction of the factors that influence Chinese college students’ willingness to use metaverse technology in online basketball courses. The results add to the theoretical understanding of the effectiveness of metaverse technology and TAM in college basketball courses. However, at this time, based on factors such as the cost-effectiveness of existing equipment, the operating mechanisms, and the management of online classrooms, widespread use may still be a few years away.

The study uses TAM in order to identify the influencing factors on college students’ willingness to learn basketball using metaverse technology, and by including flow variables in TAM, a new causal path relationship is derived. PEU, FL, and PU were found to be the key roles of UI in this model. There is a significant correlation between PU and FL, while AT and BI had a direct effect on UB. In this model, FL has a significant impact on UB and UI, and the statistical results obtained support the hypothesis model’s predictive validity. There is a significant contribution made to the combination of TAM theoretical knowledge and metaverse technology as a result of the addition of flow to the model. Furthermore, in this model, gender was found to moderate the relationship between perceived ease of use and behavioral intention, attitude and behavioral intention, and flow and attitude. Other scholars may find the results of this study useful for conducting related research.

For the developers of metaverse technology, this paper should focus more on the operability of the products. Similarly, it would be more beneficial to combine the characteristics of sports technology and provide a more targeted list of available resources in the field of physical education teaching. In terms of educators, they can create conditions, combine current technologies, explore more high-quality online teaching resources, and use advanced technologies such as metaverse more flexibly to conduct course teaching while achieving better results.

## 8. Limitations

There are a number of limitations to this study, including:

First of all, the research object is relatively simple. Metaverse technology can be applied in the field of physical education not only to college students, but also to junior high school students and social groups. The selection of research objects will be further expanded. The expansion of scientific significance in the field is important.

Second, the research subjects are ordinary college students, who have a rich scientific and technological background. Further research will investigate the actual training effect of more professional physical education students or professional players using metaverse technology to perform other sports, which will provide a higher level of technical support for the development of the metaverse technology in professional sports.

Third, in the absence of targeted resources for metaverse technology, certain deficiencies may exist in the provision of test materials. Combining metaverse’s latest products to conduct experimental research, and discussing their advantages, disadvantages, and effects, which are important to the design and promotion of metaverse’s technology and products.

## Data availability statement

The raw data supporting the conclusions of this article will be made available by the authors, without undue reservation.

## Author contributions

LR: thesis writing and data analysis. FY: thesis writing, data collection, and research method design. CG: model design and data analysis. JS: acquisition of data and editing of the work. YL: data collection. All authors contributed to the article and approved the submitted version.

## Conflict of interest

The authors declare that the research was conducted in the absence of any commercial or financial relationships that could be construed as a potential conflict of interest.

## Publisher’s note

All claims expressed in this article are solely those of the authors and do not necessarily represent those of their affiliated organizations, or those of the publisher, the editors and the reviewers. Any product that may be evaluated in this article, or claim that may be made by its manufacturer, is not guaranteed or endorsed by the publisher.
